# Pediatric patients with facial fractures: a retrospective study

**DOI:** 10.5249/jivr.v16i1.1835

**Published:** 2024-01

**Authors:** Suchetana Goswami

**Affiliations:** ^*a*^ Department of Pediatric and Preventive Dentistry, Burdwan Dental College and Hospital Burdwan, West Bengal, India.

**Keywords:** Children, Facial fractures, Maxillofacial Trauma, Mandible

## Abstract

**Background:**

Epidemiology of maxillofacial injuries vary depending on geographic location, culture and socioeconomic condition. This study assessed etiology and pattern of facial fractures in children reported at Burdwan Dental College and Hospital, Burdwan, West Bengal, India.

**Methods:**

Clinical files of 206 children with facial fractures were evaluated retrospectively. Diagnosis of fractures were confirmed by radiographic examination. Study period was from July 2018 to June 2022. Data collected were age and sex of the patient, site of fracture, and etiology of trauma. Descriptive statistics were used for statistical analysis with a P-value less than 0.05.

**Results:**

There were 127 (61.7%) males and 79 (38.3%) females with a male to female ratio of 1.6:1. Within the study sample, the majority of facial fractures (n= 146, 70.9%) belonged to 6-to-9-year age group. Overall, falls (44.2%) and motor vehicles accidents (31.5%) were the two leading causes of trauma. Mandible fracture was the most common, accounting for 72.8% of cases. 74 patients (35.9%) with facial fracture had dental injuries.

**Conclusions:**

Male predominance in facial fracture is seen. With age, frequency of facial fractures tends to increase. Falls are the main cause of facial fracture and mandible is the most common anatomic location.

## Introduction

A Facial fractures are less common in children compared to adults, especially in populations under the age of five years.^1^ Facial fractures occur in only 10% to 15% of maxillofacial trauma patients.^2,3^ However, their treatment approach represents a challenge for clinician as they are in active growing stage.^2^ The lower incidence of facial fractures in the pediatric population could be due to several reasons such as flexibility of facial bones and sutures, larger cranium, stability provided by the presence of tooth buds in the jaws, thick layers of adipose tissue covering facial bones, and underdevelopment of paranasal sinuses A search of literature indicates that nasal bones and mandible are the most commonly fractured facial bones. Fall, motor vehicles accident, sports related injury, and bicycle accidents are common modes of trauma to the facial bones in children.^1-4^ Facial fractures in younger age groups may lead to impairment of growth, psychological trauma and unaesthetic appearance of patients.^2-5^ Therefore, early diagnosis, formulation of treatment plan and intervention at appropriate time is of utmost importance to minimize complications.

Studies have indicated that incidence and pattern of facial fractures vary according to populations investigated.^4-7^ Limited data of facial fractures in children are available from the eastern part of India in general and West Bengal in particular. Understanding the etiology and pattern of facial fractures are necessary for formulating treatment and preventive strategies. This study aimed to examine etiology facial fractures occur and their patterns in children.

## Methods

**Study Subjects:**

In this retrospective study, data from pediatric patients aged 12 years or younger who were diagnosed with facial fractures and treated at the Department of Pedodontics and Preventive Dentistry, Burdwan Dental College and Hospital, Burdwan, India between July 2018 and June 2022 were analyzed. The study was approved by the Institutional Ethics committee (BDCH/IEC/072) and was conducted in compliance with the Helsinki Declaration. 

**Data collection**

The clinical records of the patients were reviewed to gather information on facial fractures in children, which were previously diagnosed based on computed tomographic examinations. The collected data from hospital records included age, sex, etiology of trauma, and the anatomic location of the fractures. Patients were divided into two age groups The causes of the facial fractures were categorized as falls, motor vehicle accidents (MVA), play, bicycle accidents, injuries from tube well handles, interpersonal violence (IPV), and animal-related injuries. Patients with incomplete records, systemic diseases, or syndromes were excluded from the study. 

**Statistical Analysis**

Statistical analysis was conducted using the Epi Info software (version 7.2; CDC, Atlanta, GA, USA). Descriptive analyses including frequency, percentage, and proportion were performed. Additionally, the significance of the findings was assessed using the chi-square tests, where applicable and a P-value of less than 0.05 was considered statistically significant.

## Results

**Age and Gender Distribution**

A total of 206 patients, with a mean age of 7.66±3.01 years (range: 1-12 years), were investigated. Among them, there were 127 males (61.7%) and 79 females (38.3%). The distribution of patients according to age and gender revealed that in the age group of 0-5 years, there were 35 males (58.3%) and 25 females (41.7%), while in the age group of 6-12 years, there were 92 males (63%) and 54 females (37%). The male-to-female ratio was 1.6:1. Among patients aged 0-5 years, the male-to-female ratio was 1.4:1, while in the 6-12 years’ age group, it was 2.5:1. The age of the patients ranged from one to 12 years, with a mean age of 7.66±3.01 years. Overall, no statistically significant difference was found between males and females (P=0.62) (Table 1).

**Table 1:** Distribution of patients according to age and gender.

Age group	Male		Female		Total		Dental injury		Fracture sites	
	N	%	N	%	N	%	N	%	N	%
**0-5 years**	35	58.3	25	41.7	60	29.1	20	27	61	26
**6-12 years**	92	63	54	37	146	70.9	54	73	174	74
**Total**	127	61.7	79	38.3	206	100	74	100	235	100

No statistically significant (P=0.62) difference was found between males and females (P=0.53). Distribution of dental injuries did not show significant difference (P=0.62).

**Dental Injuries**

 Among the patients, 29.1% were in the age group of 0-5 years, and 70.9% were in the age group of 6-12 years. Dental injuries were observed in 20 cases (27%) among 0-5-year-olds and 54 cases (73%) among 6-12-year-olds. However, the distribution of dental injuries did not show a significant difference (P=0.62) between the two age groups.

**Fracture sites according to age group**

Fracture sites were analyzed among the patients, with 26% of cases in the age group of 0-5 years and 74% in the age group of 6-12 years. The most common fracture sites were noted in the age group of 6-12 years, accounting for 174 cases (74%) compared to 61 cases (26%) in the age group of 0-5 years.

**Etiology of Fractures**

Table 2 shows the etiology of facial fractures, which varied across different age groups. Falls were the most common cause among both age groups, accounting for 63.3% of cases in the 0-5-year-olds and 36.3% in the 6-12-year-olds. Motor vehicle accidents (MVA) were also significant contributors, with 26.7% of cases in the 0-5-year-olds and 33.5% in the 6-12-year-olds.

**Table 2:** Distribution of patients with etiology of facial fractures.

Etiology	0- 5-years		6-12 years		Total	
	N	%	N	%	N	%
**Fall**	38	63.3	53	36.3	91	44.2
**MVA**	16	26.7	49	33.5	65	31.5
**Play**	3	5	24	16.4	27	13.1
**Bicycle**	2	3.3	11	7.5	13	6.3
**Tube well**	0	0	3	2.1	3	1.5
**IPV**	0	0	3	2.1	3	1.5
**Animal**	1	1.7	3	2.1	4	1.9
**Total**	60	100	146	100	206	100

Other Causes: Other notable causes of facial fractures included play-related injuries, bicycle accidents, and incidents involving animals. These causes showed varying frequencies across age groups, highlighting the diverse etiology of facial fractures among pediatric patients.

**Type of Facial Fracture**

**Figure 1:**
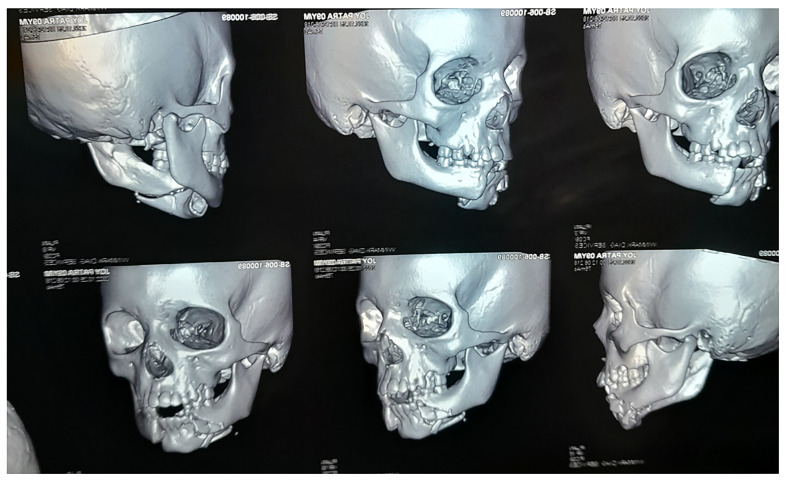
Cone beam computed tomographic image of a patient showing parasymphyseal fracture of mandible.

The type of facial fractures varied depending on the etiology and age group. Mandible fractures were the most common type among both age groups, with 52 cases in the 0-5-year-olds and 119 cases in the 6-12-year-olds. Figure 1 shows cone beam computed tomographic image of a patient with mandible fracture. Nasal fractures were also prevalent, with 8 cases in the 0-5-year-olds and 30 cases in the 6-12-year-olds.

Table 3 shows the etiology-specific analysis of facial fractures. When analyzing fracture types based on etiology, falls and motor vehicle accidents were the primary causes of mandible fractures across both age groups. However, the distribution of fracture types differed among other etiologies, such as play-related injuries and bicycle accidents.

**Table 3:** Etiology and type of facial fracture.

	Mandible		Nasal		Maxilla		Zygomatic		Orbital	
	0-5 yrs	6-12 yrs	0-5 yrs	6-12 yrs	0-5 yrs	6-12 yrs	0-5 yrs	6-12 yrs	0-5 yrs	6-12 yrs
**Fall**	33	50	5	3	0	2	0	0	0	0
**MVA**	14	36	2	17	1	10	0	7	0	3
**Play**	3	23	0	0	0	0	0	0	0	0
**Bicycle**	2	6	0	6	0	0	0	0	0	0
**Tube well**	0	2	0	1	0	0	0	0	0	0
**IPV**	0	0	0	1	0	0	0	1	0	2
**Animal**	0	2	1	2	0	0	0	0	0	0
**Total**	52	119	8	30	1	12	0	8	0	5

Yrs = years

**Distribution of Fracture Sites**

 Among the observed fracture sites, the mandible was the most commonly affected bone, accounting for 72.8% of cases. Table 4 Nasal fractures were the next most frequent, comprising 16.2% of cases, followed by fractures of the maxilla, zygomatic bone, and orbital bone.

**Table 4:** Distribution of fracture sites.

Bones	Number	Percent
Mandible	171	72.8%
Nasal	38	16.2%
Maxilla	13	5.5%
Zygomatic	8	3.4%
Orbital	5	2.1%
Total	235	100

## Discussion

In this study 206 children aged 0-12 years were included who reported to our department with facial fractures. Diagnosis of fractures were confirmed by tomographic examination. after initial examination these patients were referred to the Department of Oral and Maxillofacial Surgery of the same institute for further treatment. This institution is one of the largest tertiary care institutes of West Bengal, India. 

Zhou et al. (2020) found that the average age for maxillofacial injuries in children was 9.8 ± 5.8 years old.^8^ In the current study, patients ranged in age from one to 12 years old, with an average age of 7.66 ± 3.01 years, which is lower than the average reported by Zhou et al., but is comparable with observations of several other studies.^4,9^ In addition, results of this study show that there were two peaks of incidence of facial fractures, one in patients at the age of 6 years and the other in 10-12 years. One of the probable reasons for this could be due to child’s engagement in school at the age of 6 years. Higher frequency or occurrence of facial fractures observed in children between the ages of 10 and 12 years old could be attributed to their increased participation in sports and other outdoor activities. 

The study categorized the patients into two groups based on their age: 0-5 years, which represents preschool children, and 6-12 years, which corresponds to school-age children. Facial fractures are uncommon in children who are younger than six years old, with only approximately 1% of all facial bone fractures occurring in this age group.^1-3^ The study observed that there is a higher probability of older children, specifically those who are aged between 6 and 12 years, experiencing facial fractures. However, the distinction between the two age groups did not yield any significant statistical difference. This outcome is in line with the discoveries of numerous other studies. ^4,7-12^ The increased prevalence of facial fractures in school-age children, as opposed to preschool children, may be linked to their greater autonomy and anatomical changes that occur in their facial structures.

The study found that facial fractures occur more frequently in boys than girls, with a ratio of 1.6:1. This is in line with the results of other studies.^13-18^ Additionally, the male to female ratio increased from 1.4:1 in the 0-5 years’ age group to 1.7:1 in the 6-12 years’ age group. This increase could be due to the fact that boys are more likely to participate in physical activities at this age.

Fall and motor vehicle accidents are the primary causes of facial fractures in children globally, but different studies have reported different main causes such as road traffic accidents, falls from heights, violence, and bicycle accidents.^14-20^ In this study, falls were the leading cause of facial fractures (44.2%), followed by motor vehicle accidents (31.5%). The lower incidence of facial fractures resulting from interpersonal violence in this study could be due to the fact that the research only involved children aged 12 years and below, who are less likely to exhibit violent behavior than adolescents and adults. The variation in causes of facial fractures could also be linked to socioeconomic status, cultural practices, geographical location, and age of the population studied.

The study showed that mandible fractures were the most frequently occurring facial fracture, making up 72.8% of all fractures. The incidence of mandible fractures increased with age, which is in agreement with previous studies.^4,14,18-21^ The lower occurrence of mandible fractures in preschool-aged children may be due to the prominence of skull bones and the position of the mandible in younger children. However, in contrast, Kim et al. (2012) reported that nasal bone fractures were the most common type of fracture in maxillofacial injuries.^22^ The authors suggested that the fragility, central location, and interpersonal violence might be the reasons for the higher incidence of nasal bone fractures.^22^

Limitations of this study include its retrospective nature and small sample size. Despite some limitations, the current research contributes to the existing body of literature on facial fractures in children by shedding light on the epidemiology and trends of such injuries in a particular group. The results imply that facial fractures are more prevalent among older children, with boys being affected more frequently than girls. Furthermore, the study highlights fall and motor vehicle accidents as major causes of pediatric facial fractures, with mandible fractures being the most common. Ultimately, this research emphasizes the need for further investigation and attention to pediatric facial fractures to enhance prevention and understanding of facial injuries among this vulnerable population.
